# The Influence of Solvent Choice on the Extraction of Bioactive Compounds from Asteraceae: A Comparative Review

**DOI:** 10.3390/foods13193151

**Published:** 2024-10-02

**Authors:** Ji-Eun Lee, Jayakodyge Thilini Madushani Jayakody, Jae-Il Kim, Jin-Woo Jeong, Kyung-Min Choi, Tae-Su Kim, Chan Seo, Iman Azimi, Ji-Min Hyun, Bo-Mi Ryu

**Affiliations:** 1 Department of Food Science Nutrition, Pukyong National University, Busan 48513, Republic of Korea; leejieun6735@pukyong.ac.kr (J.-E.L.); jikim@pknu.ac.kr (J.-I.K.); 2 Department of Smart Green Technology Engineering, Pukyong National University, Busan 48513, Republic of Korea; 3 Department of Biosystems Technology, Faculty of Technology, University of Sri Jayewardenepura, Pitipana, Homagama 10208, Sri Lanka; jtmjayakody0424@gmail.com; 4 Honam National Institute of Biological Resources, 99 Gohadoangil, Mokpo-si 587262, Republic of Korea; jwjeong@hnibr.re.kr (J.-W.J.); kyungmc0111@hnibr.re.kr (K.-M.C.); taesuda@hnibr.re.kr (T.-S.K.); sc8708@hnibr.re.kr (C.S.); 5 Monash Biomedicine Discovery Institute, Department of Pharmacology, Monash University, Melbourne, VIC 3168, Australia; iman.azimi@monash.edu

**Keywords:** Asteraceae, natural product, extraction, bioactive compounds, nutraceuticals, pharmaceuticals, cosmeceuticals, physiological functions

## Abstract

While the potential of Asteraceae plants as herbal remedies has been globally recognized, their widespread application in the food, cosmetic, and pharmaceutical industries requires a deeper understanding of how extraction methods influence bioactive compound yields and functionalities. Previous research has primarily focused on the physiological activities or chemical compositions of individual Asteraceae species, often overlooking the critical role of solvent selection in optimizing extraction. Additionally, the remarkable physiological activities observed in these plants have spurred a growing number of clinical trials, aiming to validate their efficacy and safety for potential therapeutic and commercial applications. This work aims to bridge these knowledge gaps by providing an integrated analysis of extraction techniques, the diverse range of bioactive compounds present in Asteraceae, and the influence of solvent choice on isolating these valuable substances. By elucidating the interplay between extraction methods, solvent properties, and bioactivity, we underscore the promising potential of Asteraceae plants and highlight the importance of continued research, including clinical trials, to fully unlock their potential in the food, cosmetic, and pharmaceutical sectors.

## 1. Introduction

The long history of human consumption of natural products suggests that developing health functional foods and new drugs from these sources can offer safety advantages and reduce the likelihood of side effects [1]. Moreover, the diverse physiologically active substances found in natural products can potentially not only treat specific diseases but also promote overall health through mechanisms like immune system support and antioxidant activity [2]. The vast array of natural products in the world presents numerous opportunities for developing novel health functional foods and drugs, fostering a high-value industry that can boost national competitiveness and revitalize local economies [3]. For these reasons, the natural products industry warrants further development. In this context, we aim to review the physiologically active substances, efficacy, and commercialization potential specifically within the Asteraceae family.

The Asteraceae family is one of the largest families in the plant world and includes a variety of species. This species diversity suggests the possibility of containing a wide range of physiologically active substances, and plants of the Asteraceae family have the potential to be used as pharmaceutical and health functional food materials as they have been used as traditional medicines and foods for a long time [4]. This family encompasses a large variety of flowering plants, organized into about 1600 genera and more than 23,000 species. Notable species include wormwood (*Artemisia absinthium*), yarrow (*Achillea millefolium*), chamomile (*Chamaemelum nobile*), lettuce (*Lactuca sativa*), chicory (*Cichorium intybus*), artichoke (*Cynara cardunculus*), daisy (*Bellis perennis*), and dandelion (*Taraxacum officinale*) [5]. Asteraceae plants are predominantly herbaceous, with some shrubs or sub-shrubs [6]. They exhibit diverse forms, ranging from tall trees to small herbs, with varying leaf shapes and sizes. Most species are characterized by flat clusters of small, colorful flowers [4].

Plants in the Asteraceae family have traditionally been used for medicinal purposes, and modern scientific research has revealed various effects such as antioxidant, anti-inflammatory, and immune enhancement. Based on these effects, plants in this family can be used as a health functional food material and medicine [4]. Chamomile, one of the most popular teas, has traditionally been used for medicinal purposes and includes species like *Matricaria recutita* L., and *Matricaria chamomilla* [7]. *Silybum marianum*, or *milk thistle* (MT), is well-studied for its treatment of liver disease [8] and health functional foods made from milk thistle extract are available on the market. *Calendula officinalis* is recognized by the World Health Organization for its wound healing and anti-inflammatory properties and is a component of Traumeel^®^, a treatment for symptoms related to acute musculoskeletal injury such as pain and swelling [9].

Various extraction methods have been employed for the utilization of these Asteraceae species. In solvent extraction, the choice of solvent is crucial, considering factors like selectivity, solubility, and safety. A solvent with a polarity value close to that of the solute is likely to perform better according to the laws of similarity and intermiscibility. Commonly used solvents for extraction include ethanol, methanol, water, and acetone. Depending on the type of compound to be isolated and extracted, various solvents can be mixed and used [10]. Ethanol, a polar solvent, can extract a variety of polar and nonpolar compounds with high efficiency. It is particularly effective for extracting bioactive substances such as phenolic compounds, lipids and fatty acids, and terpenoids, and has the advantage of being safe for use in food and pharmaceuticals [11]. Methanol, a polar solvent with high solubility, is effective for extracting various polar compounds. Although it exhibits high extraction efficiency, caution is necessary due to its toxicity. In addition to phenolic compounds, lipids, and fatty acids, it is also used to extract anthocyanins, terpenoids, lignans, polysaccharides, proteins, and amino acids [12,13]. Acetone, a medium-polar solvent, is highly volatile, making it easy to remove the solvent after extraction. It is suitable for extracting medium-polar compounds and is particularly effective for extracting phenolic compounds and flavonoids [14]. Water is the safest and most environmentally friendly extraction method as a polar solvent. It is cost-effective and can be extracted without special equipment. However, the extraction efficiency may be low, and it is mainly used to extract water-soluble components such as phenolic compounds, anthocyanins, lipids and fatty acids, polysaccharides, saponins, vitamins, and minerals. Environmentally friendly aqueous extraction processes have received increasing attention in recent years. Hydrodistillation or vapor distillation is a method specialized in extracting volatile compounds. Distillation extraction is a method specialized for extracting volatile components. It can extract heat-sensitive compounds and is suitable for extracting volatile aroma components. However, the extraction efficiency may be low, and it has disadvantages in terms of economic feasibility [15,16]. Compared to single solvent extraction, extraction using various solvents has the advantage of increasing extraction efficiency for a specific component, selectively obtaining only a desired component, and obtaining various and high purity extracts. However, it is uneconomical in that the process becomes complicated because multiple solvents are used, which may require more time and labor [17].

Numerous studies have demonstrated that Asteraceae species possess antimicrobial, antioxidant, antifungal, anti-inflammatory, insecticidal, anticancer, liver-protective, and wound-healing properties [4,18]. and these properties have been validated via clinical trials in humans. Animal models often face limitations due to biological differences and experimental design constraints, making it difficult to directly apply findings to humans [19]. Therefore, clinical trials in humans are essential to validate the results from animal studies [20]. Extracts of plants from Asteraceae species have been administered in various human organs to prove their therapeutic and protective effects. Notably, species like *Ligularia taquetii*, *Cirsium japonicum*, and *T. officinale* have garnered attention for their diverse bioactivities, including anti-inflammatory, antioxidant, and anticancer effects [21,22,23]. These findings suggest that Asteraceae-derived compounds can be utilized as novel functional ingredients to enhance, the nutritional and health benefits of various products. By demonstrating the functions and effects of Asteraceae plant extracts through clinical trials, we can promote their utilization and commercialization.

This paper presents biological molecules extracted using different solvents from various Asteraceae plants and discusses their related biological activities for the expansion of application fields. These species include *L. taquetii*, *C. japonicum*, *C. cardunculus* L. (artichoke), *A. millefolium*, *Acmella oleracea*, *A. absinthium* (wormwood), *C. tinctorius* (safflower), *Inula crithmoides*, *Solidago virgaurea* (goldenrod), *T. officinale* (common dandelion), *Tanacetum vulgare* (tansy), *Cichorium intybus L* (chicory), *C. cardunculus*, *Cynara scolymus*, *Helianthus tuberosus* L., *Arctium lappa* (burdock), *Arctium tomentosum, Achillea cucullata, Arctium minus, Tagetes erecta, Helichrysum arenarium, Tanacetum parthenium* (feverfew), *Baccharis dracunculifolia, M recutita* (chamomile), *Ageratum conyzoides, Artemisia vulgaris, H. annuus, Eremanthus erythropappus* (Candeia), *Centaurea imperialis, Trachelospermum asiaticum, Saussurea heteromalla, Forsythia viridissima, Chromolaena odorata, Crepis vesicaria, Sonchus asper Hill, and Sonchus oleraceus* L.

**Figure 1:**
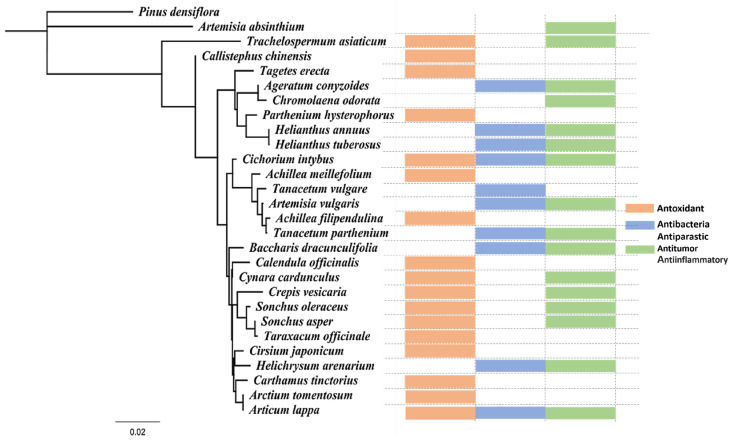
The phylogenetic tree of the Asteraceae discussed in this review. Phylogenetic trees were constructed using the rbcL region from NCBI and drawn with the Geneious Prime program. This tree encompasses the Asteraceae genus covered in this paper. Each species is classified into three main physiological activities—anti-oxidant, anti-microbial and anti-parasitic, and anti-tumor or anti-inflammatory—represented in the box plot. Red indicates antioxidant activity, blue indicates antimicrobial and anti-parasitic activity, and green indicates anti-tumor or anti-inflammatory activity. *Achillea cucullata*, *Achillea minus*, *Saura grandifolia*, *Sinulus excelsus*, *Ligularia taketi*, and *Inula crithmoides* were excluded due to the absence of rbcL gene results.

The aim of this review is to focus on the extraction of active compounds from Asteraceae plants, which have long been used in traditional medicine as teas or therapeutic agents. The review highlights the extraction solvents used and analyzes the biological properties of the extracted substances (Figure 1). It also presents the biological activities of these compounds as demonstrated through in vitro or in vivo models and clinical trials, emphasizing their potential and suitability as resources. By doing so, this review aims to showcase the applicability of Asteraceae plants in pharmaceuticals, cosmeceuticals, and functional foods, contributing to the expansion of their use and promoting commercialization.

## 2. Chemical Composition and Biological Functions of Extracts

### 2.1. Ethanol Extraction

Ethanol extraction of natural products is effective due to its broad solubility range as a neutral solvent, capable of extracting a wide variety of compounds, including both hydrophilic components soluble in water and lipophilic components like fats. Its antimicrobial properties contribute to reducing contamination risks and preserving the quality of extracts. Ethanol is technically relatively safe and economical, evaporating quickly after extraction to facilitate concentration processes. These characteristics make ethanol widely used for extracting bioactive ingredients, particularly those with high antioxidant activity, from Asteraceae plants, and it also finds applications in beverage and food manufacturing [11]. The extraction methods, physiological activities, and chemical compositions of all ethanol extracts of Asteraceae plants are summarized in Supplementary Table S1.

#### 2.1.1. Phenolic Compounds in Ethanol Extract

Asteraceae plants showed great variability in terms of phenolic compound content and profile. Phenolic compounds, including flavonoids and phenolic acids, exhibit a wide range of biological activities such as antioxidant, antimicrobial, and other effects. The antioxidant capacity of *A. lappa* isolated from 50% ethanol at 80 °C was measured using DPPH (2,2-diphenyl-1-picrylhydrazyl). The antioxidant capacity of burdock extract was 76.23 ± 0.29% in a DPPH assay and this is due to phenolic compounds, such as gallic acid (0.39%), chlorogenic acid (CGA) (43.9%), and caffeic acid (0.22%) [24].

##### Flavonoid in Phenolic Compound Group

The 70% ethanolic extracts of *A. minus* leaves and roots contained rutin and isoquercetin, genistein and nobiletin components, along with minor flavonoids such as kaempferol-3-O-rhamnoglucoside and astragalin, which have anticancer effects. Extracts of *A. minus*, at concentrations of 0.25, 2.5, 25, and 250 µg/mL, showed a selective anti-proliferative effect on K562, MCF-7, and 786-0 cancer cell lines [25].

**Figure 2:**
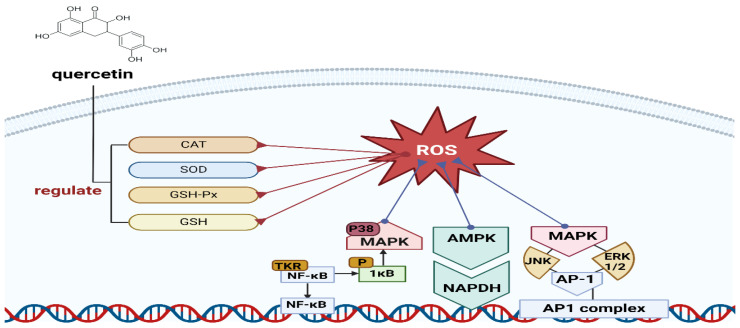
The graphical illustration for Antioxidant Mechanism of Quercetin.

Chicory leaves (*C*. *intybus* L.), which contain 112.38 mg of quercetin (Figure 2) equivalent (QE)/100 g dried weight, are a promising source with hepatoprotective properties. In plant components, phenolic chemicals and flavonoids have been shown to exhibit antimicrobial activity against *Escherichia coli* and *Pseudomonas aeruginosa* pathogens in various solvent extracts, including 70% ethanol [26].

Reactive oxygen species (ROS), produced by various factors, induce cellular toxicity and structural damage [27]. Quercetin modulates diverse oxidative defense systems and can regulate signaling pathways such as NRFB (Nuclear Respiratory Factor 1), AMPK (AMP-activated protein kinase), and MAPK (mitogen-activated protein kinase), which enhance antioxidant mechanisms. This dual regulatory role promotes antioxidant systems and controls the generation of ROS [28].

##### Phenolic Acid in Phenolic Compound Group

CGA is obtained from ethanol extracts of *L. taquetii*, often extracted at room temperature for 24 h in ethanol, yielding approximately 8.5% CGA content [21]. According to the research, this compound exhibits a spectrum of medicinal qualities, including antioxidant, antiviral, anti-inflammatory, and anti-obesity actions [29]. A research using a 3T3-L1 adipocyte model explored the anti-adipogenic properties of *L. taquetii* ethanolic extract (LTE), showing that doses of 25, 50, 100, and 200 µg/mL of LTE reduced fat accumulation by 6%, 8%, 25%, and 60%, respectively. Additionally, treatment with 50 µg/mL CGA reduced fat accumulation by 30%, with LTE at 100 µg/mL exhibiting effects similar to 50 µg/mL CGA in anti-adipogenic impact [21].

A previous study highlighted the significant presence of phenolic compounds in the leaves of Jerusalem artichoke (*H. tuberosus* L.), extracted in 70% ethanol for approximately 2 h. Total phenolic content was (266.69 ± 2.51 mg GAE/g dry extract,) and the extract displayed robust free radical scavenging capabilities. Notably, 3-O-caffeoylquinic acid (7.458%) and 1,5-dicaffeoylquinic acid (0.051%) were identified as major contributors due to their potent ability to scavenge free radicals. In particular, the DPPH radical scavenging ability of 1,5-dicaffeoylquinic acid was 11.01 ± 1.43 μg/mL, demonstrating the best antioxidant ability among the compounds tested [30].

#### 2.1.2. Lipids and Fatty Acids in Ethanol Extract

Fatty acids are abundant in the plant kingdom and exhibit structural diversity, serving as constituents of glycerolipids, sphingolipids, and extracellular lipids [31,32].

Ethanol as an amphiphilic solvent can efficiently extract lipids and fatty acids such as triglycerides, phospholipids, and free fatty acids from various plant and animal tissues. Spilanthol, was the major bioactive compound in *A. oleracea* extracted with 65% ethanol and plays an important role in its biological properties [5]. Spilanthol’s (0.103%) immunomodulatory role is attributed to its inhibition of inflammatory mediators NO, IL-1b, IL-6, and TNF-alpha, and attenuation of Cyclooxygenase-2 (COX-2) and inducible nitric oxide synthase (iNOS) expression [33].

*C. odorata L.* was extracted with 95% ethanol for 7 days and showed a high toxicity of more than 80% for all cancer cell lines at a concentration of 500 μg/mL. One of the compounds isolated from the extract was 9,12,15-octadecatrienoic acid (12.81%) [34].

##### Steroids in Lipids and Fatty Acids Group

Plants synthesize a diverse array of steroid molecules, which are significant secondary metabolites in plant physiology [35]. It has been reported that daucosterol (1%) and β-sitosterol (0.17%) are present in *A. tomentosum* extracted with 95% ethanol [25]. Daucosterol showed antidiabetic activity with an inhibition rate of 97.3% at a concentration of 200.0 mmol/mL in an α-glucosidase activity inhibition effect experiment, and the ID_50_ value (50% inhibition concentration) was found to be 30.0 mmol/mL [36]. *H. arenarium*, extracted with 70% ethanol [37], has been linked to the production of four steroid molecules: β-sitosterol, β-sitosterol-glucoside, stigmasterol, and stigmasterol-glucoside [38]. Baskar et al. employed various concentrations (5, 10, and 20 mg/kg body weight) of β-sitosterol to evaluate its antioxidant effects in their experiment. They found that β-sitosterol administered at 20 mg/kg body weight exhibited the most potent antioxidant capabilities, effectively inhibiting lipid peroxidation and enhancing the activity of antioxidant enzymes [39,40].

#### 2.1.3. Terpenoids in Ethanol Extract

Terpenoids represent the most prevalent and structurally diverse class of secondary metabolites found in plants, crucial for plant interactions with pathogens, insects, and other plants. Higher plants contain numerous terpenoid structures, exceeding 23,000 distinct compounds [41,42]. Ethanolic extracts (95% ethanol for 6 h) of A. lappa roots revealed pentacyclic triterpenoids such as ursolic acid (0.2%) and oleanolic acid (0.27%), while the sesquiterpene lactone onopordopicrin (0.59%) was extracted from Arctium lappa leaves using 95% ethanol [25,43]. *A. lappa* ethanol extract has anti-hyperlipidemic activity, suppressive effect on melanocyte and epidermal hyperproliferation, cytotoxic activity toward human pancreatic tumor, and anti-parasitic action [25].

#### 2.1.4. Minerals in Ethanol Extract

*C. intybus* L. leaves isolated with 70% ethanol contain minerals like Cr, Al, Cd, Ni, Co, and Si, while its roots are rich in Ca, P, K, and Mg. Chicory seeds exhibit a dominant mineral composition with higher concentrations of K (0.62%), Ca (1.98%), P (0.944%), Mg (0.38%), Cu (0.002%), Zn (0.006%), and Mn (0.003%) Although less studied, chicory flowers have been found to contain abundant Fe, Al, Mn, Zn, and B [26].

### 2.2. Methanol Extraction

Methanol’s low price and high extraction efficiency offer economic advantages, but it is important to consider the potential additional costs associated with safety management and compliance with environmental regulations. Although methanol is a relatively inexpensive and efficient solvent for extraction, safety and environmental concerns must be taken into account. Methanol is more polar than ethanol, allowing it to form stronger hydrogen bonds as both a hydrogen donor and acceptor within the molecule. Due to this property, methanol’s ability to extract compounds when used as a mixed solvent with water varies depending on polarity. Additionally, methanol generally has a higher solvent strength and broader solubility than water, enabling fast and efficient extraction. However, safety precautions are necessary when using methanol due to its high volatility and ability to ignite vapors. Based on its physical and chemical properties, methanol is widely used in analytical chemistry for its ability to efficiently extract various compounds such as flavonoids, terpenoids, phenolic compounds, and vitamins [12,13]. The extraction method, physiological activity, and chemical composition of all methanol extracts of Asteraceae are summarized in Supplementary Table S2.

#### 2.2.1. Phenolic Compounds in Methanol Extract

*C. japonicum* DC contains pectolinarin and 5,7-dihydroxy-6,4′-dimethoxy flavonoids. Various extraction methods have been employed to verify the physiological efficacy of *C. japonicum DC*. Some studies have used solvents mixed with methanol and butanol for extraction [44]. Pectolinarin (62.8%) and 5,7-dihydroxy-6,4′-dimethoxy flavonoids (36.5%) extracted from *C. japonicum* DC exhibited anticancer activity in S180 and H22 mice. Flavone extracts from *C. japonicum DC* showed dose-dependent tumor inhibition in S180 sarcoma mice, with the highest inhibition rate (55.77%) observed at 50 mg/kg, comparable to the positive control 5-Fu (56.77%) [45]. Two flavones, pectolinarin and 5,7-dihydroxy-6,4′-dimethoxy flavone (DDMF), were isolated and demonstrated hypolipidemic and hypoglycemic effects. In diabetic rats fed a high-carbohydrate/high-fat diet, pectolinarin and DDMF reduced plasma glucose by 24.5% and 19.6%, respectively, while an extract that is a mixture of the two, reduced it by 44.7% [44]. In another study, the anti-inflammatory properties and mechanisms of flavone cirsimaritin extracted from an ethanol extract of the aerial parts of *C. japonicum var. maackii Maxim* were examined using RAW264.7 cells and results showed That the extract and its flavonoid cirsimaritin reduced nitric oxide (NO) synthesis and the expression of inducible nitric oxide synthase in RAW264.7 cells [46].

The leaves of *C. cardunculus L*. contain higher levels of total phenolic compounds, flavonoids, and antioxidant activity compared to artichoke leaves. Key phenolic compounds identified include caffeoylquinic acid (1.56%), and Luteolin (0.407%). These compounds were extracted by stirring in 80% methanol for approximately 1 h. DPPH experiments showed antioxidant effects in the seeds (EC_50_ = 143 ± 1 μg/mL), leaf body (EC_50_ = 218 ± 11 μg/mL), head (EC_50_ = 466 ± 5 μg/mL), and leaf (EC_50_ = 1238 ± 60 μg/mL) [47].

*A. millefolium* methanolic extracts, infusions, and decoctions were evaluated for their antioxidant capabilities and potential anticancer properties, correlating with their phenolic profiles. Significant anti-oxidant substances in *A. millefolium* include flavonoids such as apigenin (1–5%) and quercetin (10–30%), as well as phenolic acids like caffeoylquinic acid. These compounds act as reducing agents, hydrogen donors, or singlet oxygen quenchers against oxidative stress-related reactive species [5].

Flower extracts of European goldenrod (*S. virgaurea* L.) and *T. vulgare* L., which were prepared with 70% methanol, showed a 50% higher total polyphenol content (about 2000 mg caffeic acid equivalent per 100 g dry matter) compared to leaf extracts [23]. Other methanol extracts of *T. vulgare* L. had high concentrations of CGA(4.365%) and rosmarinic acid (2.044%), which exhibited antioxidant and antifungal properties, especially high antioxidant ABTS activity [48].

*T. officinale* (commonly known as dandelion) has phenolic compounds with potent biological effects. Distinct polyphenols present in different parts of the plant contribute to its actions. Extracts prepared with 80% methanol for 30 min showed that phenolic fractions from dandelion petals exhibit superior antioxidant properties compared to those from dandelion leaves. Luteolin (47.25%) is among the commonly isolated compounds from dandelion. A methanol extract inhibited plasma lipid peroxidation induced by H_2_O_2_ or H_2_O_2_/Fe by approximately 50% [49].

Salicylic acid, with a content of 14,400.45 µg/g of dry weight, was identified as the predominant phenolic compound in *A. absinthium* L. (wormwood) leaf extracts prepared with methanol over a 20 h period [5]. Methanol extracts of *A. absinthium* L. had antioxidant activity according to DPPH, reducing power, and molybdenum phosphate assays [50].

The extract of *Carthamus tinctorius* L. prepared with 70% methanol showed antioxidant potential, a 94.50% inhibition of lipid peroxidation was achieved at a concentration of 20 μg/mL [51]. Sánchez et al. identified kaempferol 3-sophoroside (0.2%), Acacetin 7-O-β-glucuronide (0.8–1.4%) and salicylic acid (0.02–0.03%) as the main phenolic compounds in methanol extracts of *C. tinctorius* L. [52].

Extracts from *C. officinalis* L. and *Achillea filipendulina* Lam. leaves, extracted with 70% methanol and analyzed via high-performance liquid chromatography 3000 HPLC system with a diode array detector (DAD-3000; Dionex™, Thermo Fisher Scientific Inc., Cleveland, OH, USA), revealed various phenolic acids including 4-hydroxybenzoic acid (8.47%), vanilic acid (3.79%), and syringic acid (9.65%). Syringic acid exhibited exceptional free radical scavenging abilities and alleviated oxidative stress indicators, with scavenging capacities of 94.64% and 95.13%, respectively [53].

##### Flavonoids in Phenolic Compound Group

Various parts of plants generally contain different compounds, and each has its own bioactive ingredient. Methanol extract (*T. officialale* L.) from dandelion was reported to contain Phenolic compounds in leaves, which were tested in mice. Chicoric acid (11.7%) derived from 80% methanol extracts of dandelion petals demonstrated positive effects on lipid profile in an in vivo study, including flavonoids that inhibit the synthesis of reactive oxygen and nitrogen species [54]. The dandelion leaf extract reduced plasma triglyceride levels by 0.44 times and total cholesterol levels by 0.73 times compared to the control group. In addition, it was effective in reducing TBARS levels in the spleen and brain by about 0.8 times in vivo experiments [54].

Flavonoids of *T. erecta* were isolated by extracting with 96% ethanol for 24 h. The presence of a significant quantity of flavonoid and phenolic components in *T. erecta* led to the low IC_50_ value of 17.3 g/mL in the extracts from this plant. The dose of *T.erecta*, which contains a variety of chemicals with strong anti-predatory properties, can help treat chronic diseases such as cancer, diabetes, and cardiovascular disease [55]. While Hanifa and Yusuf reported the presence of quercetin and kaempferol in T. erecta, the LC-MS analysis of T. erecta extract by Burlec et al. identified quercetagetin glycosides as the major components [56].

Yin, et al. utilized MeOH over three days to extract *C. japonicum*, revealing high phenolic content associated with excellent hydroxyl radical scavenging and anti-oxidant activity. In particular, as a result of hydroxyl radical scavenging activity, their MeOH extract showed a concentration-dependent hydroxyl radical scavenging activity, reaching 83.5% at a concentration of 500 μg/mL. The extract also contained the flavonoid hispidulin 7-O-neohesperidoside, which was administered orally to ethanol-treated rats at daily doses of 10 mg or 20 mg per kg body weight to evaluate its anti-oxidant activity [57,58].

*S. grandifolia* stems, *Synulus excelsus* flowers, and *Aster pilosus* were found to contain the highest concentrations of three predominant phenolic compounds following methanol extraction: quercetin (1.857%), isoquercetin (4.467%), and 1,5-di-O-caffeoylquinic acid (78.25 mg/g). *Ligularia taquetii* showed peaks of isoquercitrin (0.37 mg/g) and 1,5-di-O-caffeoylquinic acid (7.825%) without quercitrin [59]. Isoquercetin, which is commonly present in *S. grandifolia* and *A. pilosus*, has the effect of up-regulating antioxidant genes and inhibiting inflammatory cytokines [60].

*H. arenarium* methanol extracts revealed 39 distinct chemicals, primarily flavonoids such as chalcone isosalipurposide, naringenin, naringenin-5-O-glucoside, and other compounds known for hepatoprotective and anti-atherosclerotic properties. These extracts were particularly rich in narirutin, naringin, eriodictyol, luteolin, galuteolin, astragalin, and kaempferol [38]. As a result of MTT analysis using RAW264.7 cells, these compounds (at concentrations of 10–100 μM) showed a cell proliferation inhibitory effect compared to the control, and ELISA analysis proved the anti-inflammatory effect of the flavonoids [61].

##### Phenolic Acids in Phenolic Compound Group

Previous studies have demonstrated that *Parthenium hysterophilus* L. was extracted using 95% methanol for 24 h. The resulting extract, obtained after evaporation under reduced pressure, has been found to exhibit a variety of physiological activities. HPLC analysis revealed the presence of various phenolic compounds in the extract, with CGA being the most abundant. In the DPPH free radical scavenging assay, the methanol extract exhibited the highest cell inhibition of 76.90% at 80 µg, with an IC_50_ value of 54.278 µg/µL. In the hemolysis assay, the highest cell inhibition of 76.90% was observed at 200 µg, with an IC_50_ value greater than 500 [62].

Studies investigating the phytochemistry of *C. scolymus* L. have identified phenolic acids as major constituents. Among these, cynarin stands out for its immunomodulatory activity. Extraction with 80% methanol for 1 h has been utilized to isolate cynarin (0.035%) from *C. scolymus* L. The extracts exhibited high anti-oxidant capacity, showing the highest DPPH radical scavenging activity with an EC_50_ value of 143 μg/mL and the highest lipid peroxidation inhibition activity with EC_50_ values of 112 and 125 μg/mL [47].

#### 2.2.2. Anthocyanins in Methanol Extract

Pelargonidin 3-O-glucoside (91%) chloride, an anthocyanin derived from *Callistephus chinensis* flowers, plays a protective role against oxidative stress induced by glutamate excitotoxicity in neuronal cells [63]. Experimental studies on cerebellar granule neurons have shown that an 80% methanol extract of *C. chinensis* reduces cell death caused by glutamate by 20%. To investigate its physiological effects, some studies have utilized methanol extraction with 0.1% hydrochloric acid [64], because adding acid lowers the pH, which increases the stability of anthocyanins, preventing their decomposition during extraction and helping to maintain the color and activity of the extracted anthocyanins [65].

Previous research has identified four anthocyanins from various Centaurea species within the Asteraceae family, including Centaurea dealbata, Centaurea montana, Centaurea nigra, Centaurea scabiosa, Centaurea simplicicaulis, and Centaurea triumfetaspera. These compounds were extracted using the method in [66]. Cyanidin 3,5-di-O-glucoside showed DPPH radical scavenging activity with an IC_50_ value of 55.2 ± 0.12 µg/mL. Moreover, cyanidin glucoside showed anticancer effects, exhibiting significant activity against LNCaP, ACHN, and MOLT-4F cell lines with IC_50_ values of 6.43 μg/mL, 18.3 μg/mL, and 6.78 μg/mL, respectively [67].

#### 2.2.3. Lipids and Fatty Acids in Methanol Extract

Various fatty acids such as linolenic acid, linoleic acid, methyl linoleate, methyl oleate, oleic acid, palmitic acid, methyl palmitate, methyl stearate, and stearic acid are present in the methanol extract of *A. lappa*. The extracts from *A. lappa* root were used at concentrations of 0.2, 0.1, and 0.02 mg/mL. In particular, methyl palmitate, methyl linoleate, and methyl linoleate showed inhibition of α-glucosidase activity of 73.4%, 66.5%, and 68.5%, indicating that they were effective in lowering blood glucose [36].

The methanolic extract (for 72 h) of *C. intybus* L. seeds includes fatty acid esters such as n-hexadecanyl hexadecanoate. Across different chicory cultivars, total fatty acid contents range from 104 to 644 mg/100 g, with α-linolenic acid comprising the majority (64%) in the leaves [26].

##### Steroid in Lipids and Fatty Acids

Sitosterol-b-D-glucopyranoside extracted with methanol from A. lappa L. showed an inhibitory effect on α-glucosidase activity. At a concentration of 11.534%, it inhibited 97.3% of α-glucosidase activity [36].

#### 2.2.4. Terpenoids in Methanol Extract

Sesquiterpene lactones are abundant in *C. intybus* L. methanol extract. The roots of this plant yield sesquiterpene lactones such as 11(S),13-dihydro-8-deoxylactucin (0–9.3%). In addition, *C. intybus* L. extract has the effect of antifungal [68].

*H. tuberosus* L. was extracted with methanol for 10 days to separate sesquiterpene lactone. Sesquiterpene lactone (0.05%) is effective its anti-oxidant, anti-cancer, and anti-fungal properties [69,70].

#### 2.2.5. Lignans in Methanol Extract

Lignans are a diverse group of naturally occurring substances synthesized via the shikimic acid biosynthetic pathway. They are widely distributed throughout the plant kingdom, and are found in the roots, rhizomes, stems, leaves, flowers, fruits, seeds, xylem, and resins of many plant species [71].

Major biologically active lignans, such as arctigenin (ATG) are abundant in seeds, roots, fruits, and leaves of *A. lappa* and *A. tomentosum*. These compounds are often isolated using methanol extraction methods. ATG, derived from *A. lappa* seeds, exhibits estrogenic effects that can reduce the risk of osteoporosis, heart disease, and menopausal symptoms. Lignans from *A. lappa* are valued for their antioxidant properties, which enable them to scavenge free radicals implicated in numerous diseases [25].

The methanol extracts of *A. lappa* have shown a wide range of biological effects, including anti-inflammatory, anti-proliferative, anti-oxidant, anti-cancer, anti-diabetic, anti-adipogenic, anti-bacterial, and UVB protective properties, with AGT being particularly significant in preventing tumor growth [72]. AGT (75.8%) found in burdock extract inhibit the COX-2 enzyme, which contributes to inflammation and irritation in wounds [25].

#### 2.2.6. Polysaccharides in Methanol Extract

Polysaccharides are complex biomolecules crucial for plants, particularly as key energy sources produced through photosynthesis [73]. They are classified based on their structural complexity: monosaccharides are simple sugars with one sugar unit, oligosaccharides have two to ten units, and polysaccharides consist of eleven or more units [74,75].

Lis et al. reported that roots (50% methanol extract) are rich in fructans like inulin [76]. In the cell walls of *A. lappa* and *A. minus* roots and leaves (extracted with methanol), various polysaccharides have been identified. These include pectic substances, rhamnogalacturonan containing neutral sugars, hemicelluloses (arabinan, arabinogalactan, galactan, xylan, and xyloglucan), galacturonic acid, glucose, galactose, arabinose, rhamnose, mannose, and cellulose. Additionally, the fruits of *A. lappa* reportedly contain arabinose, glucose, galactose, rhamnose, and raffinose [25].

#### 2.2.7. Carotene in Methanol Extract

All photosynthetic organisms produce carotenoids, which are isoprenoid metabolites and they play a variety of roles in photosynthesis, photoprotection, pigmentation, phytohormone production, and signaling in plants [77].

Nine carotenoid compounds were identified and characterized in *C. vesicaria* L., *S. Asper* L. Hill and *S. oleraceus* L. after a 15 min methanol extraction. These compounds include carotene (0.02–0.06%) (α-carotene, β-carotene, and its isomers, 9-cis-β-carotene and 13-cis-β-carotene) and xanthophyll (0.01–0.4%) (violaxanthin, neoxanthin, lutein, zeaxanthin, and β-cryptoxanthin) [78]. *S. asper* has been noted for its significant anti-oxidant and anti-inflammatory effects in previous research [79].

### 2.3. Acetone Extraction

Acetone extraction, characterized by its lower polarity and higher volatility compared to the previously mentioned ethanol and methanol extractions, is an organic solvent that readily dissolves non-polar components rather than polar substances. This solvent is particularly useful for extracting lipophilic compounds, such as vegetable oils and carotenoids, from plants. Furthermore, acetone’s high selectivity for non-polar substances allows for increased purity of the target compound. This reduces the cost of subsequent purification processes and is advantageous for the production of high-value-added products. Moreover, acetone is relatively inexpensive compared to other organic solvents, making it an economical choice [14]. The extraction method, physiological activity, and chemical composition of all acetone extracts are summarized in Supplementary Table S3.

#### 2.3.1. Polyacetylenes in Acetone Extraction

Polyacetylenes are naturally occurring compounds widely distributed in plants, characterized by structures containing two or more triple bonds [80].

From the roots of *A. lappa*, acetone extraction yielded nine sulfur-containing acetylenic compounds (0.003–0.3%): arctinone-a, arctinone-b, arctinol-a, arctinol-b, arctinal, arctic acid-b, arctic acid-c, methyl arctate-b, and arctinone-a acetate. These compounds exhibit various bioactivities, including documented antibacterial and anti-fungal properties. Polyacetylenes are known for their cytotoxic, anti-inflammatory, and antibacterial properties among other biological effects [81].

#### 2.3.2. Terpenoids in Acetone Extraction

Feverfew (*T. parthenium* L.) contains parthenolide (0.8%), a sesquiterpene lactone, and was extracted using acetone for 3 h. *T. parthenium* L. has demonstrated antimicrobial and anti-inflammatory properties. In particular, a study by Heptinstall et al. revealed that sesquiterpenelactone has a 5-HT (serotonin) secretion inhibitory function [72,82].

#### 2.3.3. Carboxylic Acids in Acetone Extraction

Most plant tissues, including edible parts like fruits, seeds, roots, leaves, and stems, contain natural carboxylic acids. The two most common acids found in plants are benzoic acid (BA) and cinnamic acid (CinA) [83].

*A. lappa* roots contain various carboxylic acids, including acetic acid, BA, butyric acid, CinA, costic acid, dodecanoic acid, hexanoic acid, (E)-3-hexenoic acid, heptanoic acid, (E)-3-heptenoic acid, 2,3-hydroxyoctanoic acid, 2-methylpropionic acid, 2-methylbutyric acid, 3-methoxybenzoic acid, nonanoic acid, nonanedioic acid, pentanoic acid, phenylacetic acid, phenylpropionic acid, propionic acid, salicylic acid, and undecanoic acid. *A. lappa* was extracted with acetone three times and contains various active phytochemicals known as hydroxycinnamic acids (86%), which possess antioxidant and free radical scavenging properties, making them effective in wound treatment and skin protection [25,81].

### 2.4. Water and Steam Extraction

The water and steam extraction method typically uses water as a solvent to extract plant materials, which are carried out at high temperatures. It uses inexpensive and readily available water, and in particular, steam extraction offers economic benefits as an energy-efficient process, which is advantageous for reducing operating costs. Although this method may not be effective for non-polar materials, it is advantageous for extracting polar compounds such as carotenoids and phenols. In particular, steam extraction, which utilizes high temperature and pressure, is highly effective in extracting volatile compounds such as essential oils [15]. However, in some cases, there are also disadvantages such as low extraction efficiency and long extraction time. Nevertheless, this approach provides the advantage of broadening the range of substances extracted from plants, reducing the presence of toxic residues and solvents compared to organic solvent extraction methods, and producing non-toxic and environmentally friendly extracts [16]. The extraction method, physiological activity, and chemical composition of all acetone extracts are summarized in Supplementary Table S4.

#### 2.4.1. Phenolic Compounds in Water and Steam Extraction

The high levels of polyphenolic compounds in artichokes are linked to their useful properties. In artichoke flower heads (*C. cardunculus* L.), the most prevalent phenolic substances (9.89%) are caffeoylquinic acid derivatives, particularly chlorogenic acid (5-O-caffeoylquinic acid), cynarin (1,5-O-dicaffeoylquinic acid), and iso-chlorogenic acid (3,5-O-dicaffeoylquinic acid) [84]. To isolate polyphenols from artichokes, they were extracted with water as a solvent in some studies [84]. Due to the high phenolic content (9.89%) of *C. cardunculus*, studies have shown that it has hepatoprotective, hypocholesterolemic, hypolipidemic, and hypoglycemic characteristics [4]. In particular, as a result of a *Cynara cardunculus* L.’ antioxidant experiment, artichoke’s water extract showed an anti-oxidant capacity of about 0.17 g TEAC/g as a result of DPPH analysis, and 50% lipid peroxidation inhibition ability [84].

##### Flavonoids in Phenolic Compounds

*T. asiaticum* was subjected to water extraction at room temperature for 24 h to investigate its biological activities. The resulting extract, which was found to contain gallic acid, exhibited notable anti-oxidant and antidiabetic properties. Water extract from *T. asiaticum* showed 80.9% scavenging activity at a concentration of 100 μg/mL in a DPPH assay, and the antioxidant capacity for fat-soluble materials showed a value of 2.43 PF [85].

#### 2.4.2. Anthocyanin in Water and Steam Extract

Cyanidin-3-O-(6″-malonyl-glucopyranoside) (0.28%), which was the most abundant anthocyanin in the acidic water extract of the leaves of *C. intybus* L. cultivars, showed antioxidant and anti-inflammatory effects. In the case of lipid peroxidation inhibitory activity, it showed inhibitory efficacy of up to 92%, and in the COX-1 and COX-2 enzyme inhibitory activity, it showed efficacy of up to 41% and 84%, respectively [86].

#### 2.4.3. Essential Oil in Water and Steam Extract

**Figure 3:**
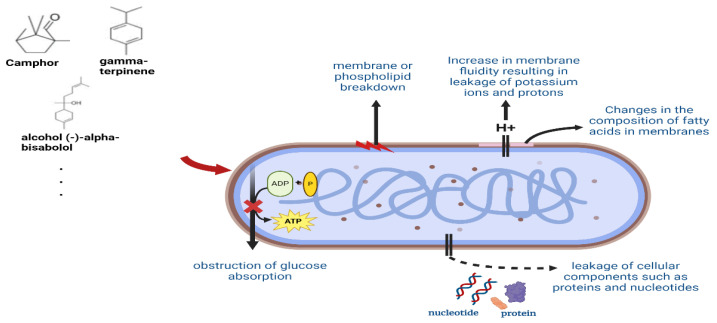
Antibacterial mechanism of essential oil.

Essential oil (Figure 3) was extracted from *T. vulgare* L. through steam distillation over a period of 3 h. This essential oil demonstrated notable anti-inflammatory, anti-oxidant, and antibacterial properties. The major compounds isolated from the essential oil derived from *T. vulgare* L. were camphor, borneol, and 1,8-cineole essential oils, which showed an anti-inflammatory effect by exhibiting an IC_50_ value of 15 μg/mL in RAW264.7 cells, and showed an anti-oxidant ability by exhibiting an average of 4.8 μg/mL of IC_50_ for DCFH oxidation. In addition, IC_50_ values of 59 μg/mL and 241 μg/mL were shown for *S. aureus* and *E. coli* strains, respectively, and by showing cytotoxicity with IC_50_ values of 0.5 μg/mL in colon cancer cells, they demonstrated the physiological activity representative of anti-bacterial and anticancer properties, respectively [87]. According to reports, the essential oil of chicory root (*C. intybus* L.) was extracted via hydro − distillation for 4 h and chicory root has 48 distinct chemicals, with camphor (20.74%), cymene (15.06%), and gamma-terpinene (13.24%) serving as the main constituents. DPPH analysis showed antioxidant activity at all three tested concentrations (0.1, 0.2, and 0.3 mg/mL), generally showing the highest antioxidant activity at 0.2 mg/mL and 0.3 mg/mL [88]. *A. conyzoides* (aerial parts), *A. vulgaris* (aerial parts), and *H. annuus* (flowers) all have essential oils which were extracted with steam distillation (about 3 h). essential oils have been shown to have antibacterial properties against *S. aureus* and *Candida* spp. The MIC (minimum inhibitory concentration) values of *A. conizoides* essential oils were 3.75 μL/mL for *S. aureus*, 7.5 μL/mL for *C. glabrata*, and 10 μL/mL for *C. albicans*. The MIC of *Artemisia bulgaris* was 2.5 μL/mL for *S. aureus*, 6.25 μL/mL for MRSA, and 5 μL/mL for *C. albicans*, *C. glabrata*, and *C. tropicalis* [55]. There are numerous benefits associated with the essential oil of *Candida* spp., including anti-inflammatory, anti-fungal, anti-bacterial, gastroprotective, and anti-cancer activities, and wound healing, and anti-nociceptive characteristics. The sesquiterpene alcohol (−)-alpha-bisabolol, which is present in this oil in high amounts, is thought to be the source of these qualities [89].

*B. dracunculifolia* is extracted with distilled water for 2 h, and substances of spathulenol (27%) and, trans-nerolidol (3,7,11-trimethyl-1,6,10-dodecatriene-3-ol) (23%) are identified. *B. dracunculifolia* essential oil shows MIC values of 0.5, 1.1, and 1.05 mg/mL for *S. aureus*, *B. cereus*, and *P. aeruginosa*, respectively, and has shown antibacterial effects against Gram (+) and Gram (−) bacteria in particular. In addition, fungi of the genus *Aspergillus versicolor*, *Penicillium funiculosum*, and *Trichoderma viride* have anti-fungal effects with MIC values of 8.43 to 16.87 mg/mL [90].

Essential oils exhibit a variety of physiological activities, primarily anti-microbial, anti-viral, anti-fungal, and insecticidal [91]. The antimicrobial ability of essential oils has received particular attention, as previous studies have evaluated the antibacterial activity in essential oils of six plants [92]. The antibacterial action of essential oils begins with inhibiting the proliferation of bacteria. The following mechanisms act as antibacterial functions: (1) decay of the phospholipid bilayer of the outer membrane of the bacteria, (2) the change in fatty acid composition, (3) the obstruction of glucose uptake, (4) the leakage of potassium and protons due to the increase in membrane fluidity, and (5) the function of enzymatic activity, or inhibiting cell lysis [93].

#### 2.4.4. Minerals in Water and Steam Extract

Petropoulos et al., reported that the 70% ethanol extract of *C. cardunculus* L. contained high levels of minerals across all plant parts, with leaves and flower heads being rich in K (0.22%) and Ca (0.18%), leaf midribs and petioles containing significant amounts of Na (0.078%), and leaf blades being notably rich in Fe (0.001%). *C. cardunculus* L. was turned to dry ash at 500 °C, and the obtained ash was dissolved in 1 N HCl to analyze the mineral components of *C. cardunculus*. Ca and Fe contents were confirmed via atomic absorption spectroscopy, and Na and K contents were confirmed via flame photometry [47].

#### 2.4.5. Vitamins in Water and Steam Extract

Plants contain a wide range of vitamins and common types of vitamins in plants are carotenoids (pro-vitamin A), ascorbate (vitamin C), and tocochromanols [94,95].

The most prevalent tocopherol observed was alpha-tocopherol, which made up roughly 86% of all tocopherols and was present in all three species (*C. vesicaria* L., *S. asper* L., and *S. oleraceus* L.). β-tocopherol was only found in S. asper, where it was found in concentrations of less than 0.1%. All three species contained sufficient levels of thiamine and little riboflavin. The extraction was performed using water containing 0.1 N HCl at 100 °C for 30 min [78]. Alpha-tocopherol was shown to be the primary vitamin in *S. oleraceus* L., which had higher tocopherol content than other wild vegetables (0.222–0.298%). According to a study by Skinner et al., antioxidant activity experiments of the tocopherol derivative show that the time (half-life) taken for half of the beta-carotene solution to oxidize is 570 h, indicating that beta-carotene delays oxidation compared to 12 h as a control group [96].

#### 2.4.6. Polysaccharide in Water and Steam Extract

*Helianthus tuberosus* L, known to have high fructose content, was boiled in water for about 5 min and then extracted in water at 60 °C for 7 h to separate inulin type fructan. Wancong Li et al. evaluated the prebiotic effects of inulin extracted from Jerusalem artichoke. The growth of probiotics in yogurt was monitored by measuring optical density (OD) and pH. The results showed that inulin with a lower DP (degree of polymerization) led to higher OD and lower pH in the culture medium after 32 h of fermentation. This indicates that inulin with a lower DP is more effectively utilized by probiotics, suggesting its superior prebiotic activity. The study suggests that harvesting Jerusalem artichoke tubers at specific times can yield inulin with varying DPs, allowing for tailored fungal applications and improved utilization in various industries [97].

### 2.5. Alternative Solvents Extraction and Multi-Solvent Extraction

Chicory leaves (*C. intybus* L.), which contain 112.38 mg of quercetin equivalent (QE)/100 g dried weight, are a promising source of flavonoids. Chicory was first extracted with methanol and ethanol. The chicory leaf and root extracts exhibited DPPH radical scavenging activity of up to 82.29% and 75.60%, respectively, with leaf extracts of up to 39 mm and root extracts of up to 31 mm (inhibitory zone diameter) upon radiation treatment of 12 kGy and 4 kGy [26,98].

*H. radiatum* was extracted by dissolving dichloromethane at room temperature for 5 min, nepetin and an O-methylated flavone nepetin was isolated. The *H. radiatum* extract showed antiviral activity with an EC_50_ value of 0.11 μg/mL against DENV-2 virus [99].

*Asteriscus maritimus* (L.) was extracted with 95% ethanol, and fractionated using petroleum ether, chloroform, ethyl acetate, and n-butanol. Luteolin, a flavonoid compound, was isolated from one of the subfractions obtained from the ethyl acetate fraction of the *A. maritimus* extract. Additionally, in mice experiments conducted with the ethanol extract of *A. maritimus*, oral administration of 100 mg/kg of the ethanol extract 1 h before the induction of oedema resulted in a 38% reduction in edema weight. In diabetic mice, the extract administered at 100 mg/kg **orally** resulted in a 14% increase in blood glutathione levels 4 h after drug administration compared to the levels observed in diabetic mice not receiving the extract [100].

*Achillea clavennae* was extracted with a solvent obtained by mixing equal volumes of ether, hexane and methanol for five days. A physiological activity effect may be expected for a sesquiterpene lactone rupicolin (2%) isolated from the extract. As a result of experiments to see the antimicrobial effect of the extract, most of the strains showed similar or higher antimicrobial activity to that of existing antibiotics, and are particularly effective against fungal strains such as *Candida albicans* and *Aspergillus niger* [101].

The extraction method, physiological activity, and chemical composition of all acetone extracts are summarized in Supplementary Table S5.

## 3. Clinical Trials

Various clinical trials (Table 1) and epidemiological studies have confirmed the beneficial health-promoting properties of Asteraceae family plants [102]. Asteraceae family plants have been traditionally utilized for medicinal purposes.

*S. chilensis* Meyen, a medicinal herb from South America, has shown effectiveness in treating tendinitis in wrist and hand muscles. Extracts were prepared by immersing the dried upper part of *Solidago chilensis* Meyen in 92.80% ethanol for 24 h. The resulting extract was incorporated into a gel cream base (5% concentration in a non-ionic cream and cellulose derivative gel). In a clinical trial involving eight participants, this formulation was applied twice daily for 21 days, leading to a significant reduction in arm pain [103].

This research focused on *A. spathulifolius* Maxim and investigated its effects on body weight, BMI, and body fat mass reduction in obese adults. Fifty kilograms of dried leaves were extracted using 50% ethanol at 60 °C for 4 h with three reflux repetitions, yielding 700 mg of extract. The extract was administered orally in capsule form to 21 obese adults once daily for 12 weeks. The results showed notable reductions in body weight, BMI, and body fat mass, suggesting its potential as a therapeutic intervention for obesity [104].

*C. rabens* is used traditionally in Taiwan for inflammatory conditions. An extraction with 95% ethanol (1:10 *w*/*v*) at 40 °C for 3 h using the dried upper portion of the plant was abtaind. The resulting extract, encapsulated and administered orally at a daily dose of 180 mg for 4 weeks to 40 participants, demonstrated improvements in skin aging parameters such as brightness, moisture content, and redness reduction, indicating potential therapeutic effects for inflammatory skin conditions [105].

*A. millefolium* L., an ancient medicinal herb from Iran, was studied in 16 patients with chronic kidney disease. Administered as flower powder (500 g) over 3 months, it showed promise in managing complications associated with chronic kidney disease by reducing plasma nitrite and nitrate concentrations compared to the placebo group [106].

*A. conyzoides*, known for its pharmacological activities, was extracted with 70% ethanol from a mixture of flowers, leaves, and stems to create a 40 mg/kg extract. This extract was applied topically four times daily to women with myiasis and ulcers caused by *Cordylobia anthropophaga*, resulting in wound healing and pain reduction within three weeks [107].

*A. lappa* L. fruit extract, known for its anti-tumor properties, was orally administered to 15 pancreatic cancer patients for 12 months. The treatment, with gradually increasing doses of arctin (starting at 68.5 mg) and arctigenin (starting at 59.4 mg), significantly extended median survival compared to untreated patients, demonstrating anti-tumor effects at the maximum dose [108].

*L. sativa*, valued for its anti-tumor and anti-inflammatory properties, was studied in 75 patients with hyperlipidemia. The extract, prepared from 50 g of crushed seeds in 250 mL aqueous-ethanolic solvent (20/80; *v*/*v*), was administered orally in capsule form for 12 weeks. The results showed that it significantly reduced triglycerides, total cholesterol, and low-density lipoprotein (LDL) levels, indicating its potential as a lipid-lowering agent [109].

*C. intybus* L., recognized for its antimicrobial and antioxidant effects, was administered to 20 patients with burns-induced liver damage. Oral administration of seed extract (10 mL three times daily) for 4 weeks resulted in reduced alanine transaminase and aspartate aminotransferase levels, suggesting hepatoprotective effects in burn-induced hepatic injury [110].

*A. wilhelmsii*, traditionally used for gastrointestinal disorders, was tested in 40 patients with active ulcerative colitis. The study involved the oral administration of *A. wilhelmsii* powder capsules twice daily for 4 weeks. The results showed no significant differences in bowel frequency or rectal bleeding severity compared to placebo, suggesting limited therapeutic efficacy of this specific formulation and treatment duration in active ulcerative colitis [111].

The study investigated the potential of *C. officinalis*, recognized for its anti-inflammatory attributes, in mitigating radiation-induced oral mucositis (OM) in 20 patients receiving radiotherapy for head and neck cancers. The treatment involved a mouthwash formulated from a 70% ethanol extraction of its flowers, administered twice daily with 5 mL each time for 7 weeks. The intervention group exhibited notably lower scores on oral mucosal assessment scales in comparison to the placebo group, suggesting the potential therapeutic value of *C. officinalis* in managing OM [58].

*C. japonicum* flower extract was obtained by extracting dried flower powder with 70% ethanol at room temperature. Twenty-three female subjects with wrinkles used a lotion containing 0.025% *C. japonicum* extract for 8 weeks. The results showed a significant decrease in the depth and volume of wrinkles around the eyes, as evaluated via 3D image analysis. Additionally, skin elasticity, measured with the R2 parameter (gross elasticity), showed significant improvement after the 8-week application period [22].

To assess the effect on primary dysmenorrhea, dried flowers of *A. millefolium* were brewed with hot water for 10 min and consumed by the participants. The severity of pain was assessed using a visual-analog scale at baseline and 1 and 2 months after treatment. The results showed a significant decrease in pain scores in the *A. millefolium* (4 g) group compared to the placebo group at both follow-up points, suggesting that *A. millefolium* may be a beneficial natural product for primary dysmenorrhea [112].

When clinical trial results of Asteraceae plants were compiled, their effects were particularly notable on inflammation-related diseases. Asteraceae plant supplementation led to symptom relief and treatment effects under several type of inflammatory disorders such as tendinitis, skin inflammation, and OM. Furthermore, Asteraceae plants revealed a critical preventive role against the underlying symptoms of the inflammatory related diseases, suggesting that Asteraceae plants may serve as potent treatment agents, such as functional nutraceuticals and cosmeceuticals for inflammation-related illnesses.

## 4. Conclusions

The Asteraceae family, with its immense biodiversity and historical significance, offers a promising avenue for addressing future challenges. These plants can contribute to food security, serving as alternative crops and novel resources to combat the food crisis. While the choice of solvent is undoubtedly crucial, optimizing the extraction conditions to suit the specific characteristics of each sample and the target bioactive compounds is equally vital in maximizing their therapeutic potential in areas such as antioxidant, antibacterial, antitumor, and anti-inflammatory activities. Clinical trials have validated their efficacy in improving health outcomes, yet their utilization remains limited.

Future research should prioritize exploring the bioactivity and chemical composition of Asteraceae plants, paving the way for expanded commercialization and applications in sectors such as pharmaceuticals, functional foods, and cosmetics. Integrating traditional knowledge with modern science can further unlock the potential of Asteraceae for developing novel therapeutics and health-promoting products. Continued investment in research and development will not only broaden the scope of Asteraceae utilization but also contribute to improving human well-being and achieving sustainable development goals.

## Figures and Tables

**Table 1:** Summary of clinical trials in human subjects.

Extract	Extract Portion	Extract Method	Target Organ	Experimental Effect	Reference
*Solidago chilensis* Meyen	Stems, leaves, petioles, and flowers	92.8% ethanol for 24 h in a percolator.	Tendonitis of flexor and extensor tendons of wrist and hand	Muscle strength improved; pain reduction effect improved	[103]
*Aster spathulifolius* Maxim	Leaves	50% ethanol at 60 °C for 4 h	Lipid	Body weight, BMI, and fat mass were significantly, decreased	[104]
*Crassocephalum rabens*	Stems, leaves, petioles, and flowers	95% ethanol (1:10 *w*/*v*) at 40 °C for 3 h	Skin	Skin brightness improved	[105]
*Achillea millefolium*	Flowers	[extraction condition unknown]	Kidney	Mean plasma concentrations of basal nitrite and nitrate decreased.	[106]
*Ageratum conyzoides* L.	Leaves, stems and flowers	70% ethanol for 4 h	Breast skin	Pain relief provided	[107]
*Arctium lappa* L.	Fruit	[extraction condition unknown]	Pancreatic cancer	Anti-tumor effect increased	[108]
*Lactuca sativa*	Seeds	80% ethanol [extraction time unknown]	Liver	LDL levels reduced, but HDL level is no significantly changed	[109]
*Cichorium intybus* L.	Seeds	Distilled water for 10 min	Liver	Liver enzymes were reduced in burn patients	[110]
*Achillea wilhelmsii*	Leaves, stems and flowers	70% ethanol at room temperature for 24 h	Colon	No significant difference between the number of bowel movements and degree of improvement in rectal bleeding	[111]
*Calendula officinalis*	Flowers	70% ethanol for 72 h	Mucous membrane of the mouth	Strength of oropharyngeal mucositis has improved	[58]
*Cirsium japonicum*	Flowers	70% ethanol at room temperature	Skin	Skin elasticity was improved, wrinkles were relieved, and aging was prevented.	[22]
*Achillea Millefolium*	Flowers	Hot water for 10 min	Lower abdomen	Alleviating menstrual pain	[112]

## Supplementary Materials

The following supporting information can be downloaded at: https://www.mdpi.com/article/10.3390/foods13193151/s1, Table S1: Ethanol extract of Asteraceae plants (extraction method, physiological activity, ingredients); Table S2: Methanol extract of Asteraceae plants (extraction method, physiological activity, composition); Table S3: Acetone extract of Asteraceae plant (extraction method, physiological activity, composition); Table S4: Water and steam extract of Asteraceae plants (extraction method, physiological activity, constituents); Table S5: Extraction of Alternative solvents from Asteraceae plants (extraction method, physiological activity, constituents).
