# Supplementary material for: The Influence of Solvent Choice on the Extraction of Bioactive Compounds from Asteraceae: A Comparative Review
Source: Foods. 2024 Oct 2;13(19):3151. doi: 10.3390/foods13193151 (PMC11475975; doi:10.3390/foods13193151)
Supplement: Supplementary file 1 [file foods-13-03151-s001.zip › foods-3194824-supplementary.pdf]

Table S1. Ethanol extract of Asteraceae plants (extraction method, physiological activity, chemical compositions).

		Plant<br>(part)	Extraction technologies	Biological functions	Chemical composition (Molecular Weight)	Compound or concentration of extract	reference
		Arctium lappa (root)	20 g sample were extracted with water-ethanol (100:100 mL) at 80 °C for 2 h.	Antioxidant, Antifungal	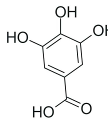 **Gallic acid** (170.12 g/mol) 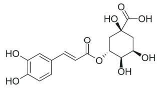 **Chlorogenic acid** (354.31 g/mol) 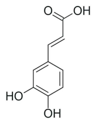 **Caffeic acid** (180.16 g/mol)	Gallic acid : 0.39 %<br>chlorogenic acid : 43.9 %<br>caffeic acid : 0.22 %<br><br>Extract dose : 0.1, 0.5, 1.0 mg/mL	[24]
Phenolic compounds	Flavonoids	*Achillea minus* (leaf)	Sample were extracted with 70 % ethanol	Anticancer	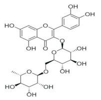 **Rutin** (610.5 g/mol) 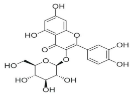 **Isoquercetin** (464.4 g/mol) 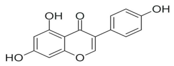 **Genistein** (270.24 g/mol) 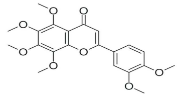 **Nobiletin** (402 g/mol)	Chemical dose: N.D.<br><br>Extract dose : 0.25, 2.5, 25, 250 µg/mL	[25]
		Cichorium intybus L. (leaf)	Sample Extracted with 70 % ethanol	Hepatoprotective properties, Antimicrobial activity	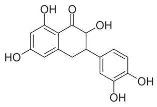 **Quercetin** (302.23 g/mol)	Quercetin : 0.11238 %<br><br>Extract dose : N.D.	[26]
	Phenolic acids	*Ligularia taquetii,* (whole plant)	5g sample were extracted with ethanol (100 mL) for 24 hours at room temperature	Antioxidant, Antiviral, Anti-Inflammatory, Anti-obesity actions	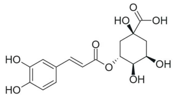 **Chlorogenic acid (354.31 g/mol)**	Chlorogenic acid : 8.5 %<br><br>Extract dose : 50 µg/mL	[21]<br>[29]

		Helianthus tuberosus L. (leaf)	100g Sample were extracted with 20 of 70 % ethanol for 2 h.	Antioxidant	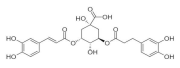 **3-O-caffeoylquinic acid** (354.31 g/mol) 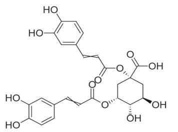 **1,5-dicaffeoylquinic acid** (516.4 g/mol)	3-O-caffeoylquinic acid : 7.458 % 1,5-dicaffeoylquinic acid : 0.051 % <br>Extract dose : 0.2-1.0 mg/mL	[30]
Lipids and fatty acids		*Acmella oleraceae* (whole plant)	Sample were extracted with 65 % ethanol.	Antioxidant, Anti-Inflammatory	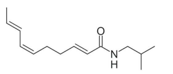 **Spilanthol** (221.34 g/mol)	Spilanthol : 0.103 % <br>Extract dose : N.D.	[5] [33]
		Chromolaena odorata L. (whole plant).	Sample (100g) were extracted with 95 % ethanol (1000 mL)	Antitumor	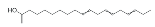 **9,12,15-Octadecatrienoic acid** (278.4 g/mol)	9,12,15-Octadecatrienoic acid : 12.81 % <br>Extract dose : 500 μg/mL	[34]
	Steroid	*Arctium tomentosum* (seed)	Dried seeds (3 kg) were extracted with hot 95 % ethanol (16 L) three times	Anti-diabetic effect	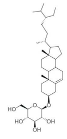 **Daucosterol** (576.8 g/mol) 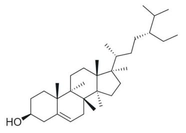 **β -sitosterol** (414.7 g/mol)	Daucosterol : 1 % β -sitosterol : 0.17 % <br>Extract dose : N.D.	[25] [36]

		Helichrysum arenarium (aerial parts)	Sample (5 kg) was extracted with 70 % EtOH	Antiinflammatory<br>Antioxidant	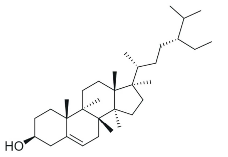<br>**β -sitosterol**<br>(414.7 g/mol)<br>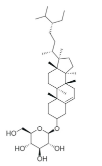<br>**β-sitosterol-glucoside**<br>(576.8 g/mol)<br>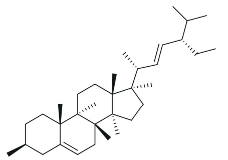<br>**Stigmasterol**<br>(412.7g/mol)<br>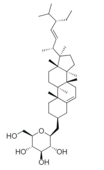<br>**Stigmasterol-glucoside**<br>(574.8g/mol)		Chemical dose: N.D.<br><br>β-sitosterol dose : 5, 10, and 20 mg/kg	[37]<br>[38]<br>[39]<br>[40]
Terpenoids		*Arctium lappa* (root)	sample (20 kg) were obtained and extracted twice with two volumes of 95 % ethanol at 50 ºC for 6 h	Anti-hyperlipidemic, Suppressive effect on melanocyte and epidermal hyperproliferation, Cytotoxic activity toward human tumor, Anti-parasitic action	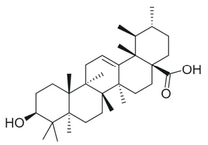<br>**Ursolic**<br>(456.7 g/mol)<br>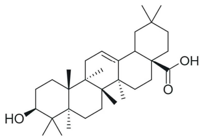<br>**β-sitosterol-glucoside**<br>(576.8 g/mol)<br>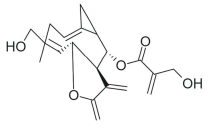<br>**Onopordopicrin**<br>(348.4 g/mol)		Ursolic : 0.2 %<br>oleanolic acid : 0.27 %<br>Onopordopicrin : 0.59 %<br><br>Extract dose : N.D.	[25]<br>[43]
Minerals		*Cichorium intybus* L.(leaf)	Extracted with 70 % ethanol	body constituents	K, Ca, P, Mg, Cu, Zn, Mn		K : 0.62 %<br>Ca : 1.98 %<br>P : 0.944 %<br>Mg : 0.38 %<br>Cu : 0.002 %<br>Zn : 0.006 %<br>Mn : 0.003 %<br><br>Extract dose : N.D.	[26]

Table S2. Methanol extract of Asteraceae plants (extraction method, physiological activity, chemical compositions)

		Plant (part)	Extraction technologies	Bbiological functions	Chemical composition (Molecular Weight)	Compound or concentration of extract	reference
Phenolic compounds		*Cynara cardunculus* L. (leaf)	Sample (1 g) and 30 mL of methanol/water (80:20 v/v, at 25 °C at 150 rpm) for 1 h	Antioxidant	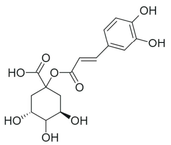 **Caffeoylquinic acid** (354.31g/mol) 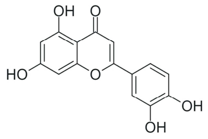 **Luteolin** (286.24 g/mol)	Caffeoylquinic acid : 1.56 % Luteolin : 0.407 %<br><br>Extract dose : 20 mg/mL	[47]
		Cirsium japonicum DC var. maackii Maxim. (whole plant)	10 g Sample were extracted with Methanol (100 mL)	Anticancer, Antidiabetic, Anti-inflammatory	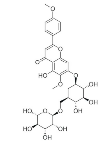 **Pectolinarin** (622.6 g/mol) 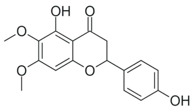 **5,7-dihydroxy-6,4'-dimethoxy flavone** (314.29 g/mol)	Pectolinarin : 62.8 % 5,7-dihydroxy-6,4'-dimethoxy flavone : 36.5 %<br><br>Extract dose : 50 mg/kg	[44] [45] [46]
		Achillea millefolium (whole plant)	1 g sample were extracted with 30 mL of methanol	Antioxidant Anticancer	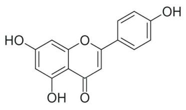 **Apigenin** (270.24 g/mol) 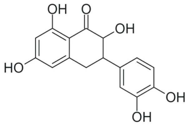 **Quercetin** (302.23 g/mol)	apigenin : 1-5 % quercetin : 10-30 %<br><br>Extract dose : N.D.	[5]
		Solidago virgaurea L., *Tanacetum vulgare L.* (leaf)	3 g sample were extracted with 70 % methanol (80 mL)	Anti-inflammatory, Anti-fungal	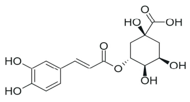 **Chlorogenic acid** (354.31 g/mol) 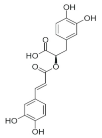 **Rosmarinic acid** (360.3 g/mol)	Chlorogenic acid : 4.365 % Rosmarinic acid : 2.044 %<br><br>Extract dose : N.D.	[23] [48]

	Taraxacum officinale (whole plant)	420 g of leaves, and 250 g of petals were extracted twice with boiling 80 % MeOH for 30 min.	Antioxidant	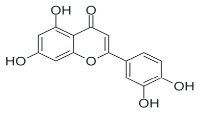<br>**Luteolin**<br>(286.24 g/mol)	Luteolin : 47.25 %<br><br>Extract dose : 10 µg/mL	[49]
	Artemisia absinthium L. (leaf)	100 g sample were extracted with Methanol for 20 h.	Antioxidant	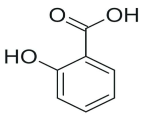<br>**Salicylic acid**<br>(138.12 g/mol)	Salicylic acid : 1.44 %<br><br>Extract dose : 20 µg/mL<br><br>Extract dose : N.D.	[5]<br>[50]
	Carthamus tinctorius L. (whole plant)	Powdered material was soaked in 70 % methanol for 3 days	Antioxidant	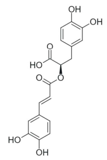<br>**Kaempferol 3-sophoroside**<br>(772.7 g/mol)<br>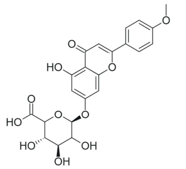<br>**Acacetin 7-O-β-glucuronide**<br>(460.4 g/mol)	Kaempferol 3-sophoroside : 0.2 %<br>Acacetin 7-O-β-glucuronide : 0.8-1.4 %<br>salicylic acid : 0.02-003 %<br><br>Extract dose : 20 µg/mL	[51]<br>[52]
	Calendula officinalis L., *Achillea filipendulina* (leaf)	0.02 g of plant material samples were extracted with 0.75 mL of 70 % methanol	Free radical scavenging, Alleviating oxidative stress indicator	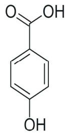<br>**4-hydroxybenzoic acid**<br>(138.12 g/mol)<br>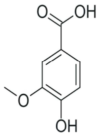<br>**Vanilic acid**<br>(168.15 g/mol)<br>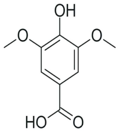<br>**Syringic acid**<br>(198.17 g/mol)	4-hydroxybenzoic acid : 8.47 %<br>Vanilic acid : 3.79 %<br>Syringic acid : 9.65 %<br><br>Extract dose : N.D.	[53]

Flavonoids	*Taraxacum officinale* L. (leaf)	Sample were extracted with methanol 80 %	Blood plasma's coagulation activity, Interference in the synthesis of reactive oxygen	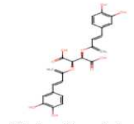<br>**Chicoric acid**<br>(474.4 g/mol)	Chicoric acid<br>: 11.7 %<br><br>Extract dose :<br>694 mg/kg	[54]
	Tagetes erecta (whole plant)	Sample (100 g) were extracted with 96 % methanol (500 ml) for 24 hours.	Antioxidant<br>Anti-cancer	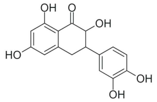<br>**Chicoric acid**<br>(474.4 g/mol)<br>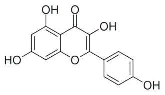<br>**Kaempferol**<br>(286.24 g/mol)	Chemical dose:<br>N.D.<br><br>Extract :<br>100 mg/mL	[55]<br>[56]
	Cirsium japonicum (aerial parts)	100 g sample were exstracted with methanol (100 mL) for 3 days at room temperature	Antioxidant,<br>Hydroxyl radical scavenging activity	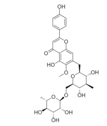<br>**Hispidulin 7-O-neohesperidoside**<br>(608.5 g/mol)	Chemical dose:<br>N.D.<br><br>Extract dose :<br>10-20 mg/kg	[57]<br>[58]
	Saussurea grandifolia<br>*Aster pilosus*<br>(flower)	Sample were extracted with MeOH at 65-75 ℃	Anti-inflammatory,<br>Antioxidant	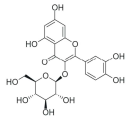<br>**Isoquercetin**<br>(464.4 g/mol)<br>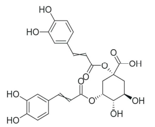<br>**1,5-O-dicaffeoylquinic acid**<br>(516.4 g/mol)<br>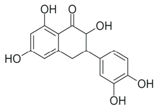<br>**Quercetin**<br>(302.23 g/mol)	Quercetin<br>: 1. 857%<br>Isoquercetin<br>: 4.467 %<br>1,5-O-dicaffeoylquinic acid<br>: 7.825 %<br><br>Extract dose :<br>40 mg/kg	[59]<br>[60]

	Helichrysum arenarium (whole plant)	800 g sample was extracted with methanol for 24 h	Hepatoprotective effect, Anti-atherosclerotic propertie	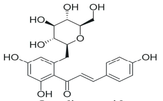<br>**Isosalipurposide**<br>(434.4 g/mol)<br>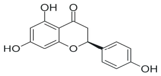<br>**Naringenin**<br>(272.25 g/mol)<br>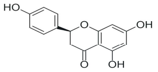<br>**Naringenin-5-O-glucoside**<br>(596.5 g/mol)<br>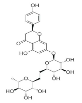<br>**Narirutin**<br>(580.5 g/mol)<br>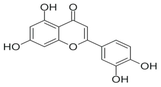<br>**Luteolin**<br>(286.24 g/mol)<br>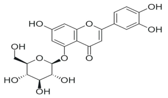<br>**Galuteolin**<br>(448.377 g/mol)<br>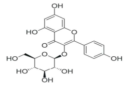<br>**Astragalin**<br>(448.4 g/mol)<br>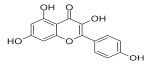<br>**Kaempferol**<br>(286.24 g/mol)<br>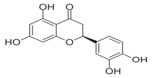<br>**Eriodictyol**<br>(302.28 g/mol)	Chemical dose: N.D.<br><br>Extract dose : 100 mM	[38]<br>[61]
Phenolic acids	*Parthenium hysterophorus* L. (flower)	24 hours extraction with methanol (95 %) solvent	Antioxidants	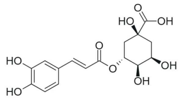<br>**Chlorogenic acid**<br>(354.31 g/mol)	Chemical dose: N.D.<br><br>Extract dose : 80-200 μg	[62]
	Cynara scolymus L. (whole plant)	Dry sample (1 g) and 30 mL of methanol/water (80:20) at 25 °C for 1 h	Immunomodulatory activity	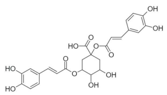<br>**Cynarin**<br>(516.4 g/mol)	Cynarin : 0.035 %<br><br>Extract dose : 20 mg/mL	[47]
Anthocyanin	*Callistephus chinensis* (flower)	50 g sample were extracted with methanol (0.01 % HCl)	Protecting neurons from oxidative stress	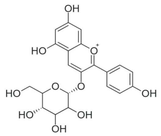<br>**Pelargonidin 3-O-glucoside**<br>(433.4 g/mol)	Pelargonidin 3-O-glucoside : 91 %<br><br>Extract dose : 0.15-0.25 %	[63]<br>[64]<br>[65]

		Centaurea species (whole plant)	Sample were extracted with either MeOH	Antioxidant, Anti-cancer	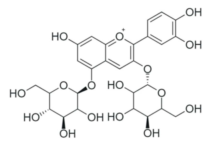<br>**Cyanidin 3,5-di-O-glucoside**<br>(611.5 g/mol)	Chemical dose: N.D.<br><br>Extract dose : 6.43- 55.2 µg/mL	[66]<br>[67]
Lipids and fatty acids	Fatty acids	*Arctium lappa* (root)	Powder samples (0.5 g) were extracted by methanol (50 mL) for 30 min.	Drop in blood sugar	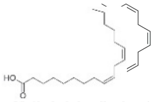<br>**Methyl alpha- linolenate**<br>(292.5 g/mol)<br>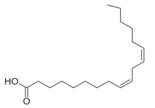<br>**Linolenic acid**<br>(280.4 g/mol)<br>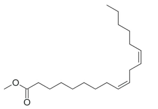<br>**Methyl linolenate**<br>(294.5 g/mol)<br>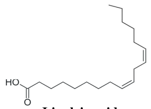<br>**Linoleic acid**<br>(278.4 g/mol)<br>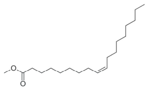<br>**Methyl oleate**<br>(296.5 g/mol)<br>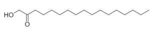<br>**Methyl palmitate**<br>(270.5 g/mol)<br>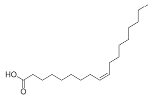<br>**Ooleic acid**<br>(282.5 g/mol)<br>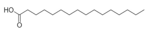<br>**Palmitic acid**<br>(256.42 g/mol)<br>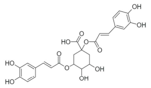<br>**Cynarin**<br>(516.4 g/mol)	Chemical dose: N.D.<br><br>Extract dose : 0.2, 0.1, and 0.02 mg/mL	[36]
		Cichorium intybus L.(seed)	Sample were extracted with methanol for 72 h	Antioxidant,<br>Anti-inflammatory properties	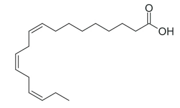<br>**α-linolenic acid**<br>(278.4 g/mol)<br>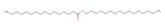<br>**n-hexadecanyl hexadecanoate**<br>**(480.8g/mol)**	Chemical dose: N.D<br><br>Extract dose : 104 to 644 mg/100 g	[28]

	Steroid	*Arctium lappa* (whole plant)	Arctium lappa (17 kg) was extracted with methanol for 12 h	Decrease glycogenolysis, Lower blood sugar levels	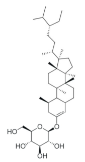 **Sitosterol-β-D-glucopyranoside** (576.8 g/mol)	Sitosterol-β-D-glucopyranoside : 11.534 % <br>Extract dose : N.D.	[36]
Terpenoids		*Cichorium intybus* L. (root)	Sample (500 mg) was extracted with methanol	Anti-diabetic	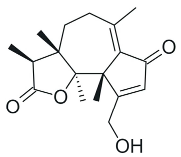 **11(S),13-dihydro-8-deoxylactucin** (262.30 g/mol)	11(S),13-dihydro-8-deoxylactucin (262.30 g/mol) : 0-9.3 % <br>Extract dose : N.D.	[68]
		Helianthus tuberosus (whole plant)	21.5 kg Sample was extracted with Methanol (180 L) for 10 days.	Antifungal, Anticancer, Antioxidants	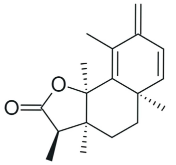 **Sesquiterpene lactone** (650.8 g/mol)	Sesquiterpene lactone : 0.05 % <br>Extract dose : 0, 0.5, 1, 5, 10, 25, 50 μg/mL	[69] [70]
Lignan		*Arctium lappa, Arctium tomentosum* (whole plant)	(3 g) Sample (pass through 50 mesh screen) was extracted for 30 min with 25 ml methanol	Antioxidant, anti-inflammatory, anti-proliferative, anti-oxidant, anti-cancer, anti-diabetic, an-ti-adipogenic, anti-bacterial, UVB protective properties	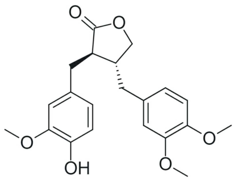 **Arctigenin** (372.4 g/mol)	Arctigenin : 75.8 % <br>Extract dose : N.D.	[25] [72]

Polysaccharide		*Arctium lappa,*<br>*Arctium minus*<br>*(*whole plant)	500 g Sample were extracted with CHCl3:MeOH (2:1) for 48 h.	Anti-inflammatory	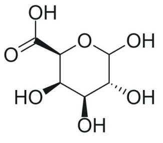<br>**Galacturonic acid**<br>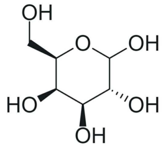<br>**Galactose**	Chemical dose: N.D<br><br>Extract dose : N.D.	[25]<br>[76]
Carotenes		*Crepis vesicaria* L.<br>*Sonchus asper* L.<br>*Sonchus oleraceus* L.<br>*(*whole plant)	Sample (0.5 g) were extracted with 7 mL of methanolfor 15 min	Antoxidant,<br>Antiinflammatory (Sonchus asper)	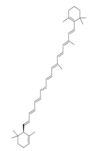<br>**α-carotene**<br>(536.9 g/mol)<br>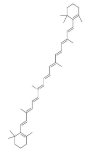<br>**β-carotene**<br>(536.9 g/mol)<br>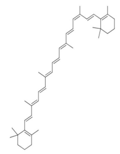<br>**9- cis-β-carotene**<br>(536.9 g/mol)<br>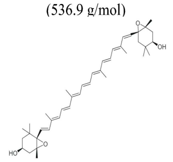<br>**Violaxanthin**<br>(600.9 g/mol)<br>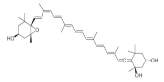<br>**Neoxanthin**<br>(600.9 g/mol)<br>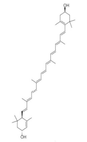<br>**Lutein**<br>(568.9 g/mol)<br>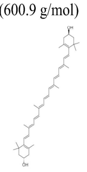<br>**Zeaxanthin**<br>(568.9 g/mol)<br>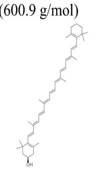<br>**β cryptoxanthin**<br>(552.9 g/mol)<br>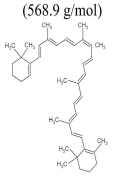<br>**13- cis-β- carotene**<br>(536.9 g/mol)	Carotene :<br>0.02 % - 0.06 %<br>xanthophyll :<br>0.01 % - 0.4 %<br><br>Extract dose :<br>12.5- 100 µg/mL	[78]<br>[79]

Table S3. Acetone extract of Asteraceae plant (extraction method, physiological activity, chemical compositions)

	**Plant (part)**	**Extraction technologies**	**biological functions**	**Chemical composition (Molecular Weight)**	**Compound or concentration of extract**	**reference**
Polyacetylenes	*Arctium lappa* (whole plant)	100 kg Sample were extracted with acetone	Antibacterial and antifungal properties, Anti-inflammatory,	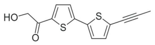<br>**Arctinone-a**<br>(262.4 g/mol)<br>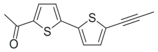<br>**Arctinone-b**<br>(246.4 g/mol)<br>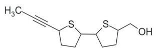<br>**Arctinol-a**<br>(234.3 g/mol)<br>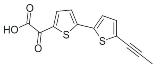<br>**Arctic Acid-b**<br>(276.3g/mol)<br>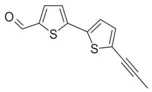<br>**Arctinal**<br>(232.3 g/mol)<br>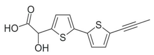<br>**Arctic Acid-c**<br>(278.4 g/mol)<br>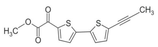<br>**Methyl Arctate-b**<br>(290.4 g/mol)<br>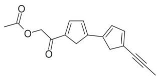<br>**Arctinone-a Acetate**<br>(304.4 g/mol)	acetylenic compound : 0.003 % - 0.3 %<br><br>Extract dose : N.D.	[81]
Terpenoids	*Tanacetum parthenium* L. (whole plant)	Sample(240 mg) was extracred with acetone (5 mL) for 3h	Anti-inflammatory, Anti-microbial	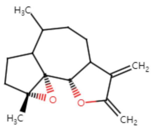<br>**parthenolide**<br>(232.3 g/mol)	Parthenolide : 0.8 %<br><br>Extract dose : 40 mg/mL	[72]<br>[82]

carboxylic acid	*Arctium lappa* (roots)	100 kg Sample were extrated three time with aceton	Wound treatment and skin protection Antioxidant	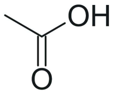<br>**Acetic acid**<br>(60.05 g/mol)<br>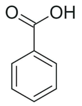<br>**Benzoic acid**<br>(122.12 g/mol)<br>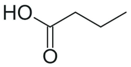<br>**Butyric acid**<br>(88.11 g/mol)<br>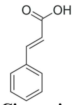<br>**Cinnamic acid**<br>(148.16 g/mol)<br>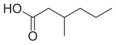<br>**(E)-3-hexenoic acid**<br>(114.14 g/mol)<br>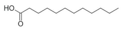<br>**Dodecanoic acid**<br>(200.32 g/mol)<br>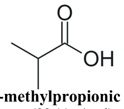<br>**2-methylpropionic acid**<br>(88.11 g/mol)<br>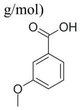<br>**3- methoxybenzoic acid**<br>(168.15 g/mol)<br>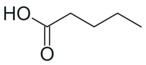<br>**Pentanoic acid**<br>**(102.13 g/mol)**<br>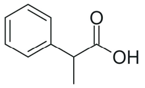<br>**Phenylpropionic acid**<br>(150.17 g/mol)<br>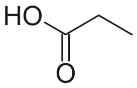<br>**Propionic acid**<br>(74.08 g/mol)<br>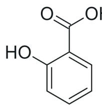<br>**Salicylic acid**<br>(138.12 g/mol)<br>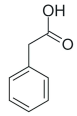<br>**Phenylacetic acid**<br>(136.15 g/mol)<br>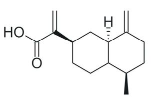<br>**Costic acid**<br>(234.33 g/mol)	Carboxylic acids : 86 %<br><br>Extract dose : N.D.	[25]<br>[81]

Table S4. Water, Steam extractof Asteraceae plants (extraction method, physiological activity, chemical compositions)

		**Plant (part)**	**Extraction technologies**	**Biological functions**	**Chemical composition (Molecular Weight)**	**Compound or concentration of extract**	**reference**
Phenolic compounds		*Cynara cardunculus* L. (flower)	Sample (1 kg) was extracted with boiling water	Antioxidant,<br>Hepatoprotective,<br>Hypolipidemic,<br>Hypoglycemic,<br>Characteristic	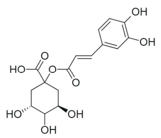<br>**Caffeoylquinic acid**<br>(354.31 g/mol)<br>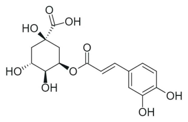<br>**5-O-caffeoylquinic acid**<br>(516.4 g/mol)<br>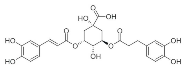<br>**3,5-O-dicaffeoylquinic acid**<br>(516.4g/mol)<br>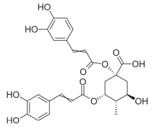<br>**1,5-O-dicaffeoylquinic acid**<br>(516.4 g/mol)<br>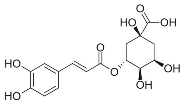<br>**Chlorogenic acid**<br>(354.31g/mol)	Phenolic compounds : 9.89 %<br><br>Extract dose : 10 mg/mL	[4]<br>[84]
	Flavonoids	*Trachelospermum asiaticum* (whole plant)	1kg Sample were extracted with Water (200 mL) for 24 h at R.T	Antioxidants,<br>Antiinflammatory	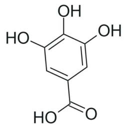<br>**Gallic acid**<br>(170.12 g/mol)	Chemical dose: N.D<br><br>Extract dose : 100 μg/mL	[85]

Anthocyanin		*Cichorium intybus* L. (leaf)	Leaves of C. intybus (100 g) were extracted with acidic water (1 % HCl, 3 × 100 mL) for 2 min at room temperature	Anti-inflammatory Antioxidant	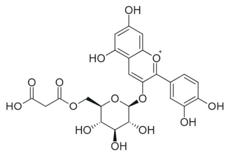 **Cyanidin-3-O-(6″-malonyl--glucopyranoside)** (535.4 g/mol)	Cyanidin-3-O-(6″-malonyl--glucopyranoside) : 0.28 % <br>Extract dose : 250 μg/mL	[86]
		Tanacetum vulgare L. (aerial parts)	Steam distillation to extract essential oils for 3 hours	Anti-inflammatory, Antioxidant, Antibacterial,	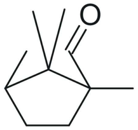 **Camphor** (128.169 g/mol) 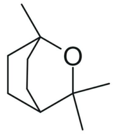 **1,8-cineole** (154.25 g/mol)	Chemical dose: N.D <br>Extract dose : 0.5-59 μg/mL	[87]
Lipids and fatty acids	Essential oils Essential oils	*Cichorium intybus* L. (root)	(20 g) Samples were hydro-distilled for 4 h.	Antioxidant	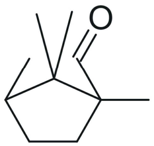 **Camphor** (128.169 g/mol) 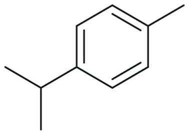 **Cymene** (134.22 g/mol) 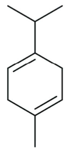 **gamma-terpinene** (136.23 g/mol)	Camphor : 20.74 % Cymene : 15.06 % gamma-terpinene : 13.24 % <br>Extract dose : 0.1, 0.2, 0.3 mg/ml	[88]
		Ageratum conyzoides, Artemisia vulgaris, (aerial parts)	Sample (100 g) were steam distillation (about 3 hours),	Antibacterial	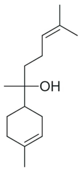 **alcohol (-)-alpha-bisabolol** (204.35 g/mol)	*Ageratum conyzoides* Extract : 3.75 μL/mL *Artemisia vulgaris* <br>Extract dose *:* 2.5 μL/mL	[55] [89]

Lipids and fatty acids		*Baccharis dracunculifolia* (aerial Parts)	Sample was extracted with distilled water for 2h	Antibacterial	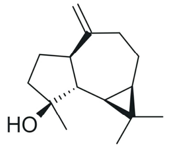<br>**Spathulenol**<br>(220.35 g/mol)<br>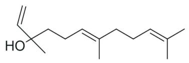<br>**Trans-nerolidol**<br>**(3,7,11-trimethyl-1,6,10-dodecatrien-3-ol)**<br>(222.37 g/mol)	Spathulenol<br>: 27 %<br>trans-Nerolidol (3,7,11-trimethyl-1,6,10-dodecatriene-3-ol)<br>: 23 %<br><br>Extract dose :<br>1 mg/mL	[90]
Minerals		*Cynara cardunculus* L. (whole plant)	Samples were subjected to dry ashing at 500 °C and extracted with 1 N HCl	body constituents	K, Ca, Na, Fe	K : 0.22 %<br>Ca : 0.18 %<br>Na : 0.078 %<br>Fe : 0.001 %<br><br>Extract dose :<br>N.D.	[47]
Vitamins		*Crepis vesicaria* L.,<br>*Sonchus oleraceus* L.<br>(whole plant)	sample (0.4 g) were extracted with 0.1 N HCl (20 ml) at 100 ° C for 30 min	Antioxidant	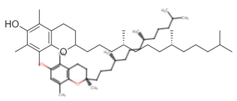<br>**Alpha-tocopherol**<br>(430.7 g/mol)<br>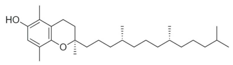<br>**Beta -tocopherol**<br>(430.7 g/mol)	Alpha-tocopherol :<br>0.222-0.298 %<br>Beta - tocopherol :<br>0-0.1 %<br><br>Extract dose :<br>N.D.	[78]<br>[96]
Polysaccharide		*Helianthus tuberosus* L. (Tuber)	Sample was boiled for 5 min and extracted 60 °C for 7 h	Prebiotic Activity	Inulin	Inulin :<br>12.21 %<br><br>Extract dose :<br>N.D.	[97]

Table S5. Alternative and Multi-solvent solvents extract of Asteraceae plants (extraction method, physiological activity, chemical compositions)

		Plant (part)	Extraction technologies	Biological functions	Chemical composition	Compound or concentration of extract	reference
Phenolic compounds	Flavonoids	*Cichorium intybus* L. (leaf)	20 g Sample was extracted with methanol and ethanol (200 mL)	Antimicrobial	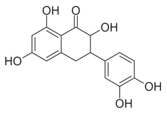<br>**Quercetin** (302.23 g/mol)	Quercetin : 0.112 %<br><br>Extract dose : N.D.	[26]<br>[98]
		Asteriscus maritimus (L.) (aerial parts)	1.5 kg Samples were extracted with 95 % ethanol, and fractionated using split ether, chloroform, ethyl acetate, and n-butanol as petroleum ether.	Anti-inflammatory, Antioxidant	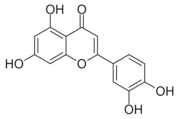<br>**Luteolin** (286.24 g/mol)	Chemical dose: N.D<br><br>Extract : 100 mg/kg	[100]
		*Helenium radiatum (*whole plant)	Sample was extracted dichloromethane (10 % w/v) for 5 min at room temperature	Antiviral	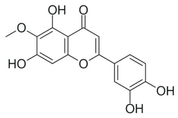<br>**Nepetin** (316.26 g/mol)	Chemical dose: N.D<br><br>Extract dose : 0.06 - 250 µg/mL	[99]
Terpenoids		*Achillea clavennae* L. (whole plant)	5 g Sample was extracted solvent obtained by mixing equal volumes of ether, hexane and methanol (100 mL) for five days	Antimicrobial	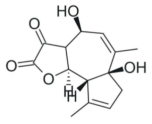<br>**Rupicolin** (262.30 g/mol)	sesquiterpene lacton : 2 %<br><br>Extract dose : 10 mg/ml	[101]
